# Aneuploidy and Confined Chromosomal Mosaicism in the Developing Human Brain

**DOI:** 10.1371/journal.pone.0000558

**Published:** 2007-06-27

**Authors:** Yuri B. Yurov, Ivan Y. Iourov, Svetlana G. Vorsanova, Thomas Liehr, Alexei D. Kolotii, Sergei I. Kutsev, Franck Pellestor, Alfia K. Beresheva, Irina A. Demidova, Viktor S. Kravets, Viktor V. Monakhov, Ilia V. Soloviev

**Affiliations:** 1 National Research Center of Mental Health, Russian Academy of Medical Sciences, Moscow, Russia; 2 Institute of Pediatrics and Children Surgery, Roszdrav, Moscow, Russia; 3 Institute of Human Genetics and Anthropology, Jena, Germany; 4 Rostov State Medical University, Roszdrav, Rostov-on-Don, Russia; 5 Institute of Human Genetics, Montpellier, France; Centre de Regulacio Genomica - Barcelona Biomedical Research Park, Spain

## Abstract

**Background:**

Understanding the mechanisms underlying generation of neuronal variability and complexity remains the central challenge for neuroscience. Structural variation in the neuronal genome is likely to be one important mechanism for neuronal diversity and brain diseases. Large-scale genomic variations due to loss or gain of whole chromosomes (aneuploidy) have been described in cells of the normal and diseased human brain, which are generated from neural stem cells during intrauterine period of life. However, the incidence of aneuploidy in the developing human brain and its impact on the brain development and function are obscure.

**Methodology/Principal Findings:**

To address genomic variation during development we surveyed aneuploidy/polyploidy in the human fetal tissues by advanced molecular-cytogenetic techniques at the single-cell level. Here we show that the human developing brain has mosaic nature, being composed of euploid and aneuploid neural cells. Studying over 600,000 neural cells, we have determined the average aneuploidy frequency as 1.25–1.45% per chromosome, with the overall percentage of aneuploidy tending to approach 30–35%. Furthermore, we found that mosaic aneuploidy can be exclusively confined to the brain.

**Conclusions/Significance:**

Our data indicates aneuploidization to be an additional pathological mechanism for neuronal genome diversification. These findings highlight the involvement of aneuploidy in the human brain development and suggest an unexpected link between developmental chromosomal instability, intercellural/intertissular genome diversity and human brain diseases.

## Introduction

The human genome seems to represent a highly dynamic and relatively instable system at interindividual and intercellular levels. The variation of chromosome numbers between individual organisms or cells of an organism is among the main types of genomic instability [1]–[4]. The biological consequences of genetic instability manifested as loss or gain of whole chromosomes (aneuploidy) usually are devastative and hallmark numerous pathological conditions in humans. Aneuploidy arisen from meiotic errors is the leading genetic cause of morbidity and mortality in humans [5]. A cascade of abnormal mitotic divisions accompanied by formation of aneuploidy is a consistent finding in virtually all cancers [6]–[8]. Moreover, abnormal functioning of mitotic machinery associated with aneuploidy formation is suggested to underlie aging [9]. Stochastic (or spontaneous) chromosomal variations in somatic cells appearing as low-level mosaic aneuploidy can be registered in all somatic cell populations. However, usually being considered insignificant, low-level somatic chromosomal mosaicism is frequently overlooked, probably, because of unapparent phenotypic effects [4], [10].

The genetic complexity of the brain is employed to explain the fascinating abilities of humans such as speech, consciousness, tool use, symbolic thought, cultural learning, and self-awareness. This can be naturally affected by different genetic and environmental factors during the intrauterine period, leading, thereby, to individual differences in brain organization and function after birth [3]. A number of attempts at the assessment of chromosome variations in the adult human brain have indicated that mosaic aneuploidy do present in the normal and diseased brain [11]–[15]. The murine brain, considered as an adequate model of human brain diseases [16], has been documented to possess aneuploid developing and adult neurons [17]. Murine aneuploid neurons are functionally active and may be integrated into the brain circuitry [18]. However, the nature, magnitude, and significance of aneuploidy in the developing and adult human brain are a matter of conjecture. To the best of our knowledge, aneuploidy in the developing human brain has not been experimentally assessed. To fill this gap in our knowledge about chromosomal (genomic) variations during human development, we have performed the study of aneuploidy in the developing human brain.

## Results

### Stochastic aneuploidy frequency in the developing brain

**Figure 1:**
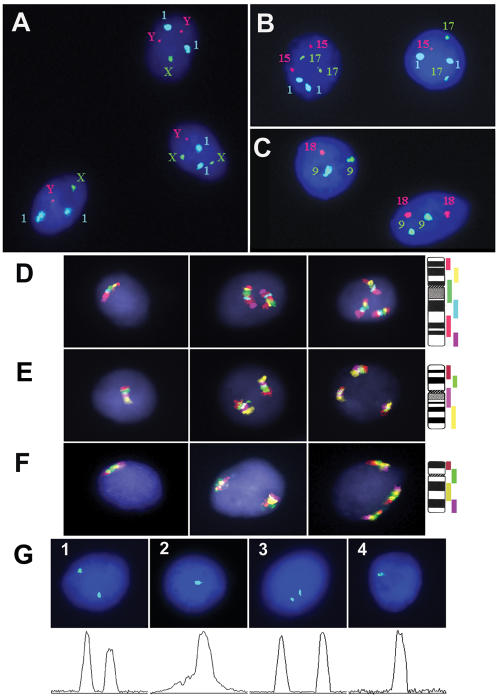
Molecular cytogenetic analysis of aneuploidy in the fetal human brain. **(A to C)**. Interphase FISH with chromosome-enumeration DNA probes: (A) two nuclei characterized by additional chromosomes Y and X and a normal nucleus; (B) a nucleus with monosomy of chromosome 15 and a normal nucleus; (C) a nucleus with monosomy of chromosome 18 and a normal nucleus. (D to G) interphase chromosome-specific MCB: nuclei with monosomy, disomy, trisomy and G-banding ideograms with MCB color-code labeling of a chromosome (from left to right), (D) - chromosome 9, (E) - chromosome 16, and (F) - chromosome 18. (G) interphase QFISH: (1) a nucleus with two signals for chromosomes 18 (relative intensities: 2058 and 1772 pixels), (2) a nucleus with one paired signal mimics monosomy of chromosome 18 (relative intensity: 4012 pixels), (3) a nucleus with two signals for chromosomes 15 (relative intensities: 1562 and 1622 pixels), (4) a nucleus with one signal showing monosomy of chromosome 15 (relative intensity: 1678 pixels).

Aneuploidy was surveyed in 12 post-mortem fetal brain samples by molecular cytogenetic techniques specially elaborated for precise identification of low-level chromosomal mosaicism at the single-cell resolution: interphase multiprobe fluorescence *in situ* hybridization (mFISH) with quantification of FISH signals (QFISH) [19], primed in situ labeling (PRINS) and interphase chromosome-specific Multicolor Banding (MCB) [15]. Interphase mFISH with arbitrary selected chromosome enumeration DNA probes for six different autosomes (chromosomes 1, 9, 15, 16, 17, 18) and the sex chromosomes (X and Y) has shown high hybridization efficiency (over 99.5%). This was considered to diminish possible misinterpretations of aneuploidy scoring (Figure 1, A to C). Analysis of more than 420,000 cells from twelve samples of the fetal brain and 85,000 chorionic villi cells was performed by interphase mFISH (Table 1). No fewer than 5000 nuclei from each brain tissue and no fewer than 1000 nuclei from chorionic villi sample were scored per chromosome. Loss of the Y chromosome was detected in 0.2% of brain cells in male fetuses, while no evidences for loss of chromosome X and simultaneously both autosomes have been obtained. One FISH signal per interphase nucleus for autosomal DNA probes was detected in 6–12% of nuclei scored in different brain tissue samples. Since quantification of FISH signals have been applied, it was possible to differ between true hypodiploid (monosomic) and euploid cells featured by associated FISH signals (a signal appearance similar to monosomy). We have determined 1.03% of the brain cells and 0.51% of chorionic cells to be true monosomic, while the most part of cells with one signal showed associated signals suggesting two homologous chromosomes in a cell (Table 1). The frequency of cells with chromosome losses in the brain was found to increase significantly versus chorionic tissues (p<0.001). The frequency of hyperdiploid cells with chromosome gains including autosomal trisomy, trisomy of the X chromosome (female fetuses), disomy of the X chromosome (male fetuses), and tetrasomy in the fetal brain was similar to that in chorionic tissue (0.42 and 0.46%, respectively, p = 0.08). Polyploidy (tetraploidy) was detected in 0.04% of fetal brain cells and in 0.06% of chorionic cells (p = 0.026). Multiple numerical chromosome imbalances involving more than one chromosome pair in one cell (i.e. simultaneous losses or gains of several non-homologous chromosomes) were not registered at all. Simultaneous gain of the chromosome X and chromosome Y was detected in 0.02% of cells in one brain sample only. We have observed significant difference in the frequency of aneuploidy (losses + gains) between the brain and chorionic villi (1.45 and 0.98, respectively, p<0.001; Table 1). The mean stochastic (or background) aneuploidy rate involving individual chromosomes (M), standard deviation (SD), the threshold levels (M+3SD) for chromosome losses and gains were calculated (Table 2). Stochastic chromosome losses and gains (the rates calculated without outliers) in fetal brain cells were registered with the mean frequency 0.91% (SD 0.37) and 0.34% (SD 0.19) per individual chromosome pair, respectively. Cut-of levels (M+3SD) were determined as 0.44–3.38% for losses and 0.40–2.68% for gains affecting different chromosomes (Table 2).

**Table 1:** Comparison of aneuploidy frequency in the fetal brain cells and chorionic villi cells detected by interphase mFISH analysis (12 fetuses analyzed).

Tissue	Number of cells scored	Normal diploid cells	Aneuploid Cells (loss)	Aneuploid cells (gain)	Aneuploid cells (loss+gain)	Polyploid cells
Brain	424674	418356 (98.5%)	4361 (1.03%)	1774 (0.42%)	6135 (1.45%)	183 (0.04%)
Chorion	85123	84241 (98.97%)	438 (0.51%)	392 (0.46%)	830 (0.97%)	52 (0.06%)
P-values			P<0.001	P = 0.08	P<0.001	P = 0.026

Eight arbitrary selected chromosomes (chromosomes 1, 9, 15, 16, 17, 18, X and Y) were analyzed for each fetus. No less than 5000 cells were scored for each chromosome for the brain tissue and 1000 cells for chorionic tissue.

**Table 2:** Stochastic aneuploidy frequency (%) involving chromosome loss and gain and the average chromosome instability index (losses and gains summed per individual chromosome pair) in the human fetal brain

Chromosome; Number of scored cells (n)	Mean (SD) without outliers	Threshold level (M+3SD)	Min(Outliers), Max (Outliers)	Mean with outliers (SD)
Chromosome 1 loss and gain; n = 60745	1.04 (0.44) and 0.28 (0.11)	2.36 and 0.61	0.3; 1.7 and 0.1; 0.4	
Chromosome 9 loss and gain; n = 60922	0.69 (0.46) and 0.20 (0.14)	2.07 and 0.62	0.2; 1.6 and 0.1; 0.6	
Chromosome 15 loss and gain; n = 60554	0.97 (0.56) and 0.20 (0.10)	2.65 and 0.40	0.3; 2,2 (6.2) and 0.1; 0.4	1.41 (1.60)
Chromosome 16 loss and gain; n = 60714	1.08 (0.57) and 0.23 (0.13)	2.79 and 0.62	0.3; 2.0 and 0.1; 0.4	
Chromosome 17 loss and gain; n = 60558	0.75 (0.35) and 0.18 (0.10)	1.8 and 0.48	0.3; 1.3 and 0.1; 0,4	
Chromosome 18 loss and gain; n = 60791	0.92 (0.64 and 0.33 (0.16)	2.84and 0.81	0.3; 2.2(6.5) and 0.1; 0.5	1.39 (1.72)
Chromosome X loss and gain; n = 26436 (female fetuses); n = 33954 (males fetuses)	1.60 (0.59) and 0.73 (0.65)	3.37 and 2.68	0.8; 2.2 (5.4) and 0.0; 2.0	2.36 (1.77) 0.90 (0.84)
Chromosome Y loss and gain; n = 33954	0.20 (0.08) and 0.58 (0.70)	0.44 and 2.68	0.0; 0.3 and 0.1; 2.0 (5.8)	1.32 (2.07)
Mean (SD), loss and gain; n = 424674	0.91 (0.37) and 0.34 (0.19)			1.03 (0.59) and 0.42 (0.38)
Average Chromosomal Instability Index; (Loss+Gain); n = 424674	1.25			1.45

### Confined chromosomal mosaicism in the developing brain

**Figure 2:**
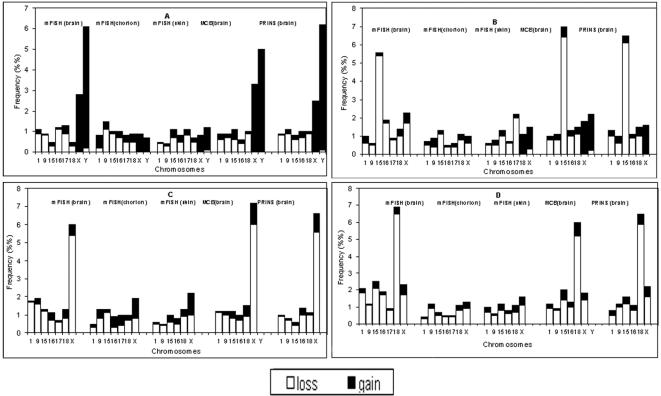
The frequency of chromosome losses and gains in the fetal human tissues exhibiting chromosomal mosaicism confined to the fetal brain. Aneuploidy frequency involving chromosomes 1, 9, 15, 16, 17, 18, X and Y was determined by interphase mFISH, MCB and PRINS techniques. (A) demonstration of selective chromosome X and chromosome Y gains, (B) demonstration of selective chromosome 15 loss, (C) demonstration of selective chromosome X loss, and (D) demonstration of selective chromosome 18 loss.

Four fetal brain samples were characterized by chromosome-specific aneuploidy inasmuch as the rate of aneuploidy involving single chromosome pair in these samples was significantly higher than the cut-off level (Figure 2, Table 2). The chromosomal mosaicism confined to the fetal brain was referred to aneuploidy manifested as (i) chromosome X gain (2.8% versus 1.2% of cells in chorion; p = 0.004) and chromosome Y gain (5.9% versus 1.8% of cells in chorion; p<0.001), (ii) chromosome X loss (5.4% versus 1.1% of cells in chorion; p<0.001), (iii) chromosome 15 loss (6.2% versus 1.2% of cells in chorion; p<0.001); (iv) chromosome 18 loss (6.5% versus 3.2% of cells in chorion; p<0.001). These outliers were detected in the fetal brain only and were observed neither in chorionic cells nor in skin fibroblasts of the same fetuses as was additionally documented by QFISH, PRINS, and MCB (Figure 1, G, and Fig. 2). Only one fetus had low-level chromosome specific mosaicism confined to the chorionic villi, i.e. trisomy of chromosome 18 in 2.3% (cut-off level is 0.8%), while only 0.4% of fetal brain cells had trisomy 18 (p<0.001). The application of PRINS and MCB for evaluation of random fluctuation of aneuploidy rate (chromosomes 1, 9, 15, 16, 18, X and Y) scoring more than 180,000 cells has confirmed the results obtained by interphase mFISH (Figure 1, D to F). Average chromosome instability quantitative index was calculated as 1.25 (without outliers) and 1.45 (with outliers) in the fetal brain (Table 2). Since molecular cytogenetic studies using DNA probes for randomly selected arbitrary chromosomes have exhibited similar pattern of involvement in autosomal aneuploidy and multiple aneuploidy has not been detected, we assumed that extrapolating the data towards the entire chromosome set (or entire genome) would not be exceedingly speculative. Cumulative frequency of stochastic aneuploidy or the overall percentage of aneuploid cells in the developing brain, calculated for 22 pairs of autosomes and two sex chromosomes, is, therefore, 30% (without outliers). Taking into account the existence of chromosome-specific brain-confined mosaic aneuploidy, the overall percentage of aneuploid cells in the developing human brain tends to approach 35%. Average chromosome instability quantitative index in chorionic tissues and fetal skin was estimated as 0.98% and 0.82% corresponding to the overall aneuploidy frequency of 24% and 19%, respectively. Studying over 600.000 individual neural cells, we have concluded that the developing human brain exhibits increased level of stochastic aneuploidy and is frequently affected by chromosome-specific mosaicism confined to the brain. Therefore, the developing human brain has mosaic nature as both euploid (∼70%) and aneuploid (∼30%) neural cells are present.

## Discussion

Somatic mosaicism makes an important contribution to genetic and phenotypic variation among humans. Somatic chromosomal mosaicism producing intercellular genomic variations simultaneously involving from hundreds to thousands genes possesses the potential to produce the most dramatic changes of cellular physiology and behavior [10]. Interphase FISH technique is known as a powerful approach to assess intercellular chromosome variations at the single cell level [3], [4]. However, FISH is affected by potential artefactual problems that might interfere with analysis of chromosome gain and loss in a cell. To avoid considering FISH artifacts as false-positive chromosome losses or gains, we have introduced two independent approaches: QFISH and interphase chromosome-specific MCB, allowing accurate definition of the normal thresholds of aneuploidy in human somatic cells [15], [19].

The first documented tissue-specific chromosomal mosaicism in normal human pregnancies was confined placental mosaicism exclusively expressed in extraembryonic tissues, which is not considered a chromosomal abnormality directly affecting fetus [20]. The developing human brain is, probably, the first embryonic tissue demonstrating confined chromosomal mosaicism. The chromosomal mosaicism confined exclusively to embryonic somatic tissue has the potential to possess primary effect on the intrauterine development. Therefore, chromosomal mosaicism confined to the fetal brain can be considered a possible cause of alterations in prenatal brain development, leading to fetal loss or abnormal brain functioning after birth.

Most postmitotic cells populating the brain are formed from neuronal and glial precursor cells originated from the embryonic neural stem cells. Chromosomal segregation defects during mitosis can occur in rapidly proliferating stem cells and, thereafter, in neuronal/glial progenitor cells with limited self-renewal ability. Aneuploid proliferating progenitor cells can be precursors of aneuploid neurons and glial cells. Previous studies indicate that the mean rate of aneuploidy per chromosome pair is, probably, ranged between 0.1 and 0.7% with the overall frequency of aneuploidy approaching 10% in the adult human brain [14], [15] and is exactly three times less than during the early brain development. Coincidental with early developmental processes of proliferation, migration and differentiation, 20–50% of fetal brain cells normally undergo programmed cell death (PCD) [21]. Ontogenetic decrease of aneuploidy rate indicates that the clearance of aneuploid cells in the developing brain could be one of the main functions of PCD. This agrees with the speculation assuming PCD as a mechanism for neural cell quantity and, probably, quality control during the development of the CNS. The data obtained suggest a new role of PCD in the developing human CNS that could be the protection of the brain against devastating consequences of developmental genomic instability. It was hypothesized that genetic and epigenetic alterations in the apoptotic machinery may result to failed clearance of abnormal neuronal and glial cells leading, thereby, to the persistence of aneuploid cells throughout ontogeny [4]. Furthermore, increased developing chromosome instability affecting neuronal and glial cells could be a possible factor predisposing to brain tumors, which are highly incident among children. Although these inferences may appear speculative, the link between PCD, developmental chromosomal instability (aneuploidy) and normal/abnormal CNS development seems to exist.

Most part of neurons and glial cells in the adult human brain are generated during intrauterine period of life. The adult human brain consists of 95–100 billions of neurons and as much as one trillion of glial cells [22]. A typical human mature neuron has approximately 5,000–200,000 synapses [3]. Aneuploid neurons are functionally active and may be integrated into the brain circuitry [18]. As aneuploidization is a pathogenic mechanism that alters gene expression, the presence of chromosomally abnormal neurons in the neuronal network should negatively affects neuron-neuron or neuron-glia interaction and, therefore, the normal functioning of the brain. Therefore, chromosome variations in the developing human brain should be ultimately involved in the pathogenesis of common mental disorders. This speculation is in accordance with the experimental data suggesting that Alzheimer's disease, schizophrenia and autism are likely to associate with increased level of aneuploidy and polyploidy in different tissues, including, probably, the brain [11], [12], [23]. In conclusion, mosaic aneuploidy affecting the developing brain represents a specific type of intercellular genomic variations contributing to the generation of the neuronal diversity and to genetic diseases of the brain [3], [4], [24].

## Materials and Methods

### Sample Preparations

Post-mortem brain tissue of 12 human fetuses (gestational age 8–11 weeks) were provided by the Brain and Tissues bank of Medical University of Rostov-on-Don, Russia. The Ethical Committees of Institutions involved approved all interventions, and tissue was collected with proper consent. The written consents of mothers of the fetuses were obtained. The study using human fetal tissues performed according to the ethical guidelines of medical research that are in accordance with Russian Federation laws and the rules for manipulation with human embryonic and fetal neuronal tissues accepted by European Commission [25]. Ultrasonic and neuropathological examination found no evidences of disease or developmental abnormalities. A cytogenetic study of chorionic villy cells demonstrated normal karyotypes in all fetuses. The telencephalic regions of the fetal brains were subjected to analysis. Additionally, autopsy tissues of chorionic villi and fetal skin of these specimens were selected for molecular cytogenetic studies. The tissue were collected and stored at −70°C. The processing of the fetal brain tissue for molecular cytogenetic analysis is described in detail elsewhere [26]. Shortly, the samples were processed through disaggregating by homogenizer, treatment with acetic acid solution (45–60% w/v), and fixation with methanol/acetic acid mixture (3:1). The suspensions obtained were dropped onto wet slides similarly to the preparation of metaphase chromosome spreads. The slides were then dried overnight at room temperature, dehydrated through ethanol series and processed for FISH.

### Multiprobe Fluorescence in Situ Hybridization (mFISH)

Interphase multiprobe fluorescence in situ hybridization (mFISH) assay using chromosome enumeration DNA probes was used [12], [14]. Chromosome 1 (D1Z1)-, 9 (D9Z1)-, 15 (D15Z1)-, 16 (D16Z3)-, 17(D17Z1)-, 18 (D18Z1)-, X (DXZ1)-, and Y (DYZ3)-specific probes labeled either by FluorX (green), Cy3 (red), or diethylaminocoumarine (blue) were used for multiprobe FISH as described in details earlier (14). The following probe combinations have been used: (1+X+Y), (1+9+16), (1+15+17), (1+9+18). Epifluorescence microscopy analysis was performed using a Leitz Orthoplan microscope (Leica Mikroskopie und Systeme, Leitz-Wetzlar; Wetzlar, Germany) as well as filter sets for 4,6-diamidino-2-phenylindole (DAPI), fluorescein isothiocyanate, Spectrum Orange or Cy3. For each tissue sample and each chromosome enumeration probe no fewer than 5000 interphase nuclei were scored.

### Quantitative FISH technique (qFISH)

Since the specifity of chromosome positioning in interphase nuclei do not allow the precise identification of chromosome loss due to FISH signal associations, the fraction of nuclei demonstrating one hybridization signal was analyzed additionally by a quantitative FISH technique [15] to discriminate single paired signals from true single signal (true monosomy). Each interphase nuclei showing one hybridization signal was captured for quantification of the signal intensity. The numerical values of the signal relative intensity in the nuclei with one (true monosomy and two associated signals) and two separate signals (disomy) were compared. The relative intensity of FISH signals was obtained by digital capturing of microscopic image by CCD camera (Cohu, 4910 series, Cohu Inc., San Diego, CA), LG-3 grayscale scientific PCI frame grabber (Scion Corp., NIH, Frederick, MD), and measuring signal intensity by Scion Image Beta 4.0.2 (Scion Corporation, National Institute of Health, Frederick, MD) acquired from www.scioncorp.com (accessed 12/07/2001). The quantification of FISH signals from each digital image was processed by the macros supplied by the manufacturer.

### Primed in Situ Labeling (PRINS)

An alternative molecular cytogenetic techniques allowing aneuploidy estimation-primed in situ labeling (PRINS) for chromosomes 1, 9, 15, 16, 18, X and Y was applied to analyze the phenomenon of confined chromosomal mosaicism revealed by mFISH. The PRINS labeling was performed by using chromosome-specific primers for the centromeric alpha-satellite DNA motifs of aforementioned chromosomes. PRINS protocol and primers used were described in details earlier [27].

### Multicolor Banding (MCB)

Finally, to achieve the highest resolution in aneuploidy scoring, a multicolor banding (MCB) assay generated on the interphase nuclei [28], shown to be among the most efficient cytogenetic approach for studying chromosomes in the human brain [12], was applied. High resolution multicolour-banding (MCB) patterns were generated with human microdissection-derived probe-sets specific for chromosomes 1, 9, 15,16, 18, X and Y. MCB is a three to five color FISH approach producing a reproducible fluorochrome profile along the chromosomal axis of interphase and metaphase chromosomes. The methodology was described ealier by Dr. Thomas Liehr and coworkers [28]. Epifluorescence microscopy analysis was performed using an Axioplan II microscope (Zeiss, Jena, Germany) equipped with a CCD camera (Sony), an HBO 100 mercury lamp, as well as filter sets for 4,6-diamidino-2-phenylindole (DAPI), diethylaminocoumarine (DEAC), fluorescein isothiocyanate, Spectrum Orange, Texas Red, and Cy5. Images were captured and analyzed using the ISIS digital imaging system (MetaSystems, Altlussheim, Germany).

### Data Analysis

No fewer than 5000 nuclei were scored per chromosome and per sample in multiprobe FISH and PRINS studies and no fewer than 3000 nuclei were assessed per sample per chromosome for interphase chromosome-specific MCB assay for the brain tissue samples, and 1000 nuclei for fetal skin and chorionic villi cells. The mean frequencies of aneuploidy (M), standard deviations (SD), thresholds levels for gains and losses for outliers of the respective chromosomes (M+3SD), and statistical significance were determined. The chromosome instability quantitative index (the fraction of cells with abnormal chromosome number accounted per individual chromosome pair) was calculated according to Lengauer and associates [29].

## References

[1] Feuk L, Carson AR, Scherer SW (2006). Structural variation in the human genome.. Nature Rev Genet.

[2] Sharp AJ, Cheng Z, Eichler EE (2006). Structural variation of the human genome.. Annu Rev Genomics Hum Genet.

[3] Muotri AR, Gage FH (2006). Generation of neuronal variability and complexity.. Nature.

[4] Iourov IY, Vorsanova SG, Yurov YB (2006). Chromosomal variations in mammalian neuronal cells: known facts and attractive hypotheses.. Int Rev Cytol.

[5] Hassold T, Hunt P (2001). To err (meiotically) is human: the genesis of human aneuploidy.. Nat Rev Genet.

[6] Lengauer C, Kinzler KW, Vogelstein B (1998). Genetic instabilities in human cancers.. Nature.

[7] Rajagopalan H, Lengauer C (2004). Aneuploidy and cancer.. Nature.

[8] Cimini D, Degrassi F (2005). Aneuploidy: a matter of bad connections.. Trends Cell Biol.

[9] Ly DH, Lockhart DJ, Lerner R, Schultz PG (2000). Mitotic misregulation and human aging.. Science.

[10] Youssoufian H, Pyeritz RE (2002). Mechanisms and consequences of somatic mosaicism in humans.. Nat Rev Genet.

[11] Yang Y, Geldmacher DS, Herrup K (2001). DNA replication precedes neuronal cell death in Alzheimer's disease.. J Neurosci.

[12] Yurov YB, Vosrtikov VM, Vorsanova SG, Monakhov VV, Iourov IY (2001). Multicolor fluorescent in situ hybridization on post mortem brain in schizophrenia as an approach for identification of low-level chromosomal aneuploidy in neuropsychiatric diseases.. Brain Dev.

[13] Rehen SK, Yung YC, McGraig MP, Kaushal D, Yang AH (2005). Constitutional aneuploidy in the normal human brain.. J Neurosci.

[14] Yurov YB, Iourov IY, Monackhov VV, Soloviev IV, Vorsanova SG (2005). The variation of aneuploidy frequency in the developing and adult human brain revealed by an interphase FISH study.. J Histochem Cytochem.

[15] Iourov IY, Liehr T, Vorsanova SG, Kolotii AD, Yurov YB (2006). Visualization of interphase chromosomes of the human brain by multicolour banding (MCB).. Chromosome Res.

[16] Watase K, Zoghbi HY (2003). Modeling brain diseases in mice: the challenges of design and analysis.. Nat Rev Genet.

[17] Rehen SK, McConnel MJ, Kaushal D, Kingsbury MA, Yang AH (2001). Chromosomal variation in neurons of the developing and adult mammalian nervous system.. Proc Natl Acad Sci USA.

[18] Kingsbury MA, Friedman B, McConnel MJ, Rehen SK, Yang AH (2005). Aneuploid neurons are functionally active and integrative into brain circuitry.. Proc Natl Acad Sci USA.

[19] Iourov IY, Soloviev IV, Vorsanova SG, Monakhov VV, Yurov YB (2005). An approach for quantitative assessment of fluorescence in situ hybridization (FISH) signals for applied human molecular cytogenetics.. J Histochem Cytochem.

[20] Kalousek DK, Dill FJ (1983). Chromosome mosaicism confined to the placenta in human conceptions.. Science.

[21] Rakic S, Zecevic N (2000). Programmed cell death in the developing human telencephalon.. Eur J Neurosci.

[22] Williams W, Herrup K (1988). The control of neuron number.. Ann Rev Neurosci.

[23] Yurov YB, Vorsanova SG, Iourov IY, Demidova IA, Beresheva AK (2007). Unexplained autism is frequently associated with low-level mosaic aneuploidy.. J Med Genet.

[24] Iourov I, Vorsanova SG, Yurov YB (2006). Intercellular genomic (chromosomal) variations resulting in somatic mosaicism: mechanisms and consequences.. Curr Genomics.

[25] Boer GJ (1994). Ethical guidelines for the use of human embryonic or fetal tissue for experimental and clinical neurotransplantation and research.. J Neurol.

[26] Iourov IY, Vorsanova SG, Pellestor F, Yurov YB (2006). Brain tissue preparations for chromosomal PRINS labeling.. Methods Mol Biol.

[27] Pellestor F, Andreo B, Puechberty J, Lerfort G, Sarda P (2006). PRINS as an efficient tool for aneuploidy assessment in human oocytes and preimplantation embryos.. Methods Mol Biol.

[28] Liehr T, Heller A, Starke H, Rubtsov N, Trifonov V (2002). Microdissection based high resolution multicolor banding for all 24 human chromosomes.. Int J Mol Med.

[29] Lengauer C, Kinzler KW, Vogelstein B (1997). Genetic instabilities in colorectal cancers.. Nature.

